# Clinical and pathological characteristics of congenital neuroblastoma in 15 Chinese infants: a retrospective analysis from a single-center

**DOI:** 10.3389/fmed.2026.1854318

**Published:** 2026-09-07

**Authors:** Li’ou Zhu, Ying Wu, Wen Zhang, Qiong Feng, Dengyan Chen, Lili Liu

**Affiliations:** Department of Pathology, Wuhan Children’s Hospital (Wuhan Maternal and Child Healthcare Hospital), Tongji Medical College, Huazhong University of Science and Technology, Wuhan, China

**Keywords:** adrenal tumor, clinicopathology, congenital neuroblastoma, infant, molecular markers, NSE

## Abstract

**Objective:**

This study aims to elucidate the clinicopathological characteristics, molecular profiles, and prognostic outcomes of congenital neuroblastoma in Chinese infants.

**Methods:**

A retrospective single-center analysis was conducted on 15 pathologically confirmed congenital neuroblastoma cases in China from 2017 to 2025. Clinical, imaging, pathological features and FISH for *MYCN* amplification and *KMT2A* rearrangement were evaluated.

**Results:**

The cohort included 15 patients with a male-to-female ratio of 8:7. Eight cases were detected prenatally and seven within 28 postnatal days. Most tumors (12/15) arose in the adrenal gland. Preoperative serum NSE elevation was more sensitive than VMA. All patients underwent complete resection; one received adjuvant chemotherapy and one relapsed. No *MYCN* amplification or *KMT2A* rearrangement was found.

**Conclusion:**

In this single-center Chinese cohort, congenital neuroblastoma showed favorable biological behavior. Complete resection achieved good outcomes. Combination of NSE and imaging examinations is promising for postoperative prognostic evaluation and recurrence surveillance in these pediatric patients.

## Introduction

Neuroblastoma represents the most prevalent extracranial solid malignant neoplasm in children younger than 2 years of age. A distinct subset, congenital neuroblastoma, is characterized by an unpredictable clinical trajectory, which may include spontaneous regression, differentiation into benign lesions, or malignant progression accompanied by metastasis (1). These variable outcomes complicate clinical diagnosis, therapeutic decision-making, and prognostic evaluation. The present study conducted a retrospective analysis of clinical data from 15 patients, aiming to delineate their clinicopathological and molecular biological features. The findings are intended to inform and enhance diagnostic accuracy and treatment approaches.

## Materials and methods

This study retrospectively collected cases of congenital neuroblastoma in pediatric patients who were admitted and treated at Wuhan Children’s Hospital between August 2017 and October 2025. The inclusion criteria were as follows: (1) diagnosis established within 28 days postpartum; (2) patients underwent surgical tumor resection, either complete or palliative; (3) postoperative pathological confirmation of neuroblastoma; (4) availability of comprehensive clinical, imaging, surgical, and pathological data; and (5) informed consent obtained from the patients’ guardians for follow-up and data analysis.

All tumor specimens were fixed in 10% neutral buffered formalin, routinely dehydrated, and embedded in paraffin. Serial sections of 3.5 μm thickness were prepared and stained with hematoxylin and eosin (H&E) for routine histopathological evaluation. Immunohistochemical analyses were conducted to assess the expression of markers including phox2B, Synaptophysin (Syn), Neurofilament (NF), Neuron-Specific Enolase (NSE), and Chromogranin A (CgA). In selected cases, fluorescence *in situ* hybridization (FISH) was performed to evaluate the status of *MYCN* and/or *KMT2A* (11q23) genes. All pathological slides were independently reviewed and diagnoses confirmed by two senior attending pathologists.

## Results

### Clinical data and laboratory findings

**Figure 1:**
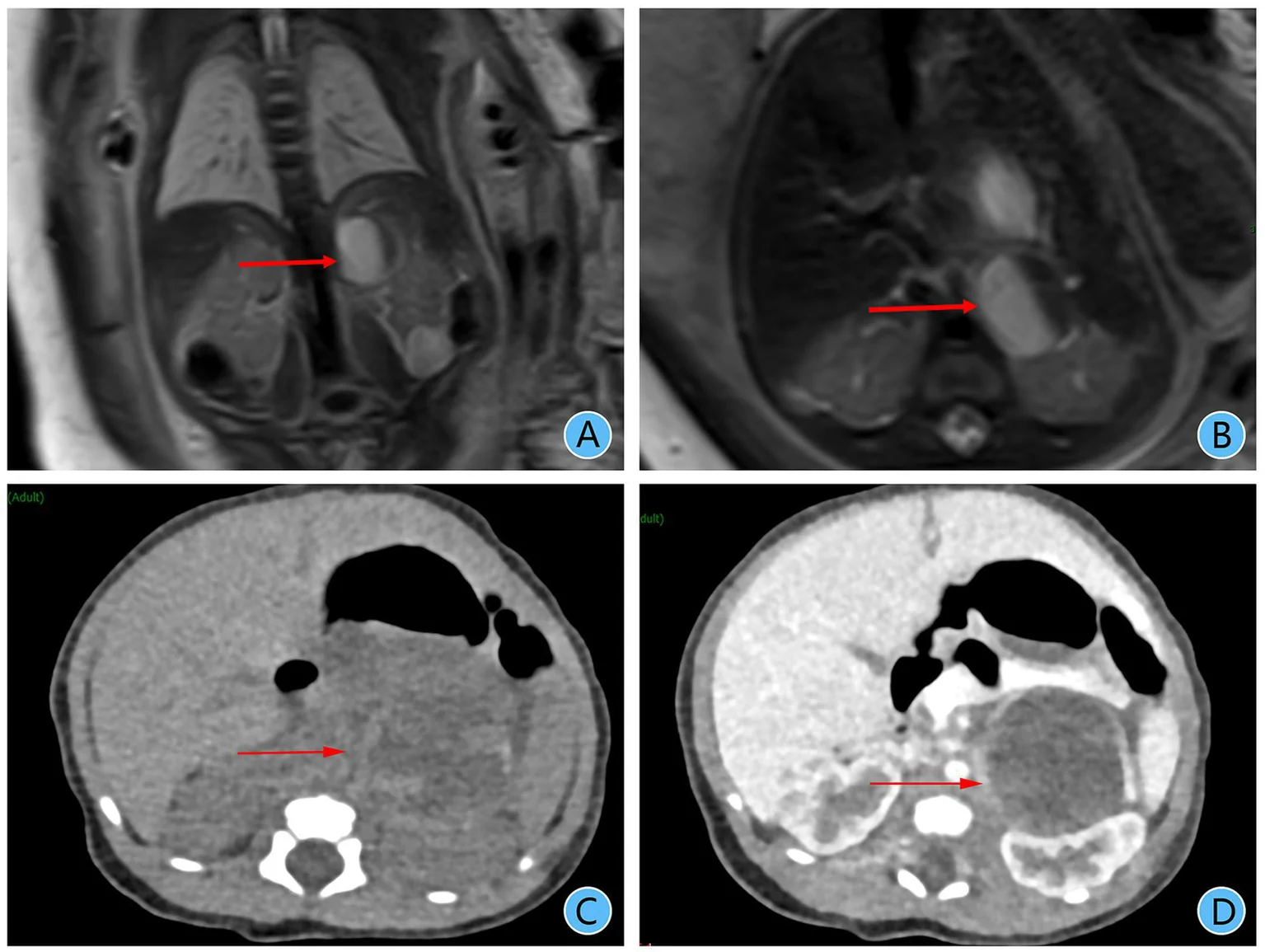
**(A)** Coronal view of a T2-weighted MRI sequence obtained during the third trimester of pregnancy, revealing a round lesion with mixed signal intensity and a fluid level located in the left fetal adrenal region; **(B)** Axial view of the same T2-weighted MRI sequence, similarly demonstrating a round lesion with mixed signal intensity and a fluid level in the left fetal adrenal area; **(C)** Postnatal non-contrast CT scan depicting a predominantly low-density lesion with mixed density in the corresponding region, containing multiple patchy areas of high density; **(D)** Postnatal contrast-enhanced CT image illustrating the same predominantly low-density mixed-density lesion with multiple patchy high-density foci, which exhibit no significant enhancement.

The cohort comprised 15 pediatric patients, with a male-to-female ratio of 8:7. Prenatal ultrasonography identified an abdominal mass in eight cases between 32 and 39 weeks of gestation (mean gestational age: 36 weeks). Seven cases were diagnosed within 28 days after birth, with two presenting due to jaundice and five due to symptoms such as persistent crying prompting imaging studies. Tumor localization was predominantly in the adrenal glands in 12 cases (10 left-sided, 2 right-sided), while three cases involved the mediastinum, neck, and retroperitoneum, respectively, with clear demarcation from the adrenal glands and kidneys (Table 1). All 15 tumors showed imaging features consistent with neuroblastoma, including 11 solid lesions and 4 cystic lesions. In Case 3, prenatal MRI using T2-weighted imaging (T2WI) at 33 weeks gestation revealed a round, heterogeneous mass with fluid–fluid levels located in the left adrenal gland (Figures 1A,B). Following delivery of the infant, CT scans of the same lesion displayed a mainly hypodense mass with mixed density, containing several patchy areas of increased density (Figures 1C,D).

**Table 1:** Clinicopathological, molecular and follow-up data of 15 patients.

Patient No.	Age of onset	Age of treatment	Gender	Anatomic location	Size (cm)	Histological diagnosis	INSS	MKI	MYCN	Follow-up (months)	Treatment	Recurrence
1	Prenatal (39 weeks)	24 days	Female	Left adrenal gland	4.7 × 3.8 × 2.7	Neuroblastoma, poorly differentiated	I	Low	MYCN amplification ‘heterogeneous’	1	Surgery	No
2	7 days	13 days	Female	Right adrenal gland	2.1 × 1.7 × 0.3	Neuroblastoma, poorly differentiated	I	Low	Gain	3	Surgery	No
3	Prenatal (33 weeks)	5 days	Female	Left adrenal gland	4.7 × 3.4 × 0.8	Neuroblastoma, poorly differentiated	I	Low	Gain	lost	Surgery	No
4	Prenatal (37 weeks)	65 days	Male	Left adrenal gland	7.5 × 6 × 6	Neuroblastoma, poorly differentiated	I	Low	Gain	7	Surgery	No
5	Prenatal (35 weeks)	1 year	Male	Left adrenal gland	2.7 × 1.7 × 1.2	Neuroblastoma, poorly differentiated	I	High	Gain	10	Surgery	No
6	Prenatal (32 weeks)	21 days	Female	Left adrenal gland	3.5 × 3 × 0.5	Neuroblastoma, poorly differentiated	I	Low	Normal	3	Surgery	No
7	6 days	16 days	Female	Left adrenal gland	4.2 × 3.3 × 1.6	Neuroblastoma, poorly differentiated	I	Low	Gain	47	Surgery	No
8	11 days	53 days	Male	Left adrenal gland	3 × 2.5 × 2	Neuroblastoma, poorly differentiated	I	Low	Gain	60	Surgery	No
9	28 days	34 days	Female	retroperitoneal	2.2 × 1.3 × 1	Neuroblastoma, poorly differentiated	I	Intermediate	Normal	61	Surgery	No
10	3 days	4 days	Male	Left adrenal gland	8 × 6 × 5	Neuroblastoma, poorly differentiated	IVS	Intermediate	/	71	Surgery + adjuvant chemotherapy	No
11	Prenatal (39 weeks)	4 h	Female	Left adrenal gland	5.5 × 4 × 3.5	Neuroblastoma, poorly differentiated	I	Intermediate	/	76	Surgery	No
12	Prenatal (36 weeks)	42 days	Male	Right adrenal gland	5 × 4 × 2.7	Neuroblastoma, poorly differentiated	IIB	Intermediate	/	68	Surgery	No
13	Prenatal (39 weeks)	1 days	Male	Left adrenal gland	4 × 3 × 2	Neuroblastoma, poorly differentiated	I	Low	/	lost	Surgery	No
14	3 days	11 days	Male	Left posterior mediastinum	5 × 4 × 1	Neuroblastoma, poorly differentiated	I	Low	Gain	6	Surgery	Relapsed after 6 mouth
15	0 days	1 year and 10 mouth	Male	Neck	4.5 × 2.5 × 1.5	Ganglioneuroblastoma, nodular	IIB	Low	Gain	1	Surgery	No

Laboratory analyses showed that preoperative serum neuron-specific enolase (NSE) testing was performed in 12 of the 15 patients (80%), including 11 cases with elevated NSE levels and 1 with normal levels (Table 2). Postoperative NSE data were collected from 10 of these 12 patients. All exhibited varying degrees of NSE reduction, and five patients’ levels returned to the normal range. All children underwent complete surgical resection of the tumors. Postoperative follow-up ranging from 47 to 71 months was performed in 12 patients with postoperative NSE follow-up data. Among them, NSE levels returned to the normal reference range in 7 patients and remained persistently elevated in five. The five patients with elevated postoperative NSE levels were divided into two groups. Three presented transient elevation, with levels normalizing within 3 months. Cases 8 and 9 showed recurrent fluctuations in NSE levels on serial follow-up examinations. Although their NSE levels remained high for a long time, no tumor recurrence was observed throughout follow-up, and their levels gradually approached the normal range at 47 and 49 months postoperatively, respectively. Of the seven patients with normalized postoperative NSE levels, one developed tumor recurrence, with the primary tumor located in the left posterior mediastinum. Quantitative vanillylmandelic acid (VMA) testing was completed preoperatively in 13 of the 15 patients, with elevated results detected in two patients.

**Table 2:** Pre-operative and post-operative NSE and VMA values.

Patient No.	Pre-operation NSE (ng/ml) reference range: 0 ~ 16.3	Post-operation NSE (ng/ml) reference range: 0 ~ 16.3	Pre-operation VMA (mg/24 h) reference range: 0.0 ~ 12
1	28.85	13.14	2.01
2	20.08	12.51	0.26
3	51.75	/	0.31
4	76.92	11.58	16.49
5	16.26	16	0.85
6	19.54	15.13	0.12
7	28.6	27.2	1.32
8	65.66	19.14	1.43
9	37.32	17.83	Negative
10	/	36.83	59.5
11	103. 9	39.17	Negative
12	/	/	Negative
13	65.95	/	/
14	42.78	13.15	0.77
15	/	11.39	/

### Pathological examination

#### Gross pathology

**Figure 2:**
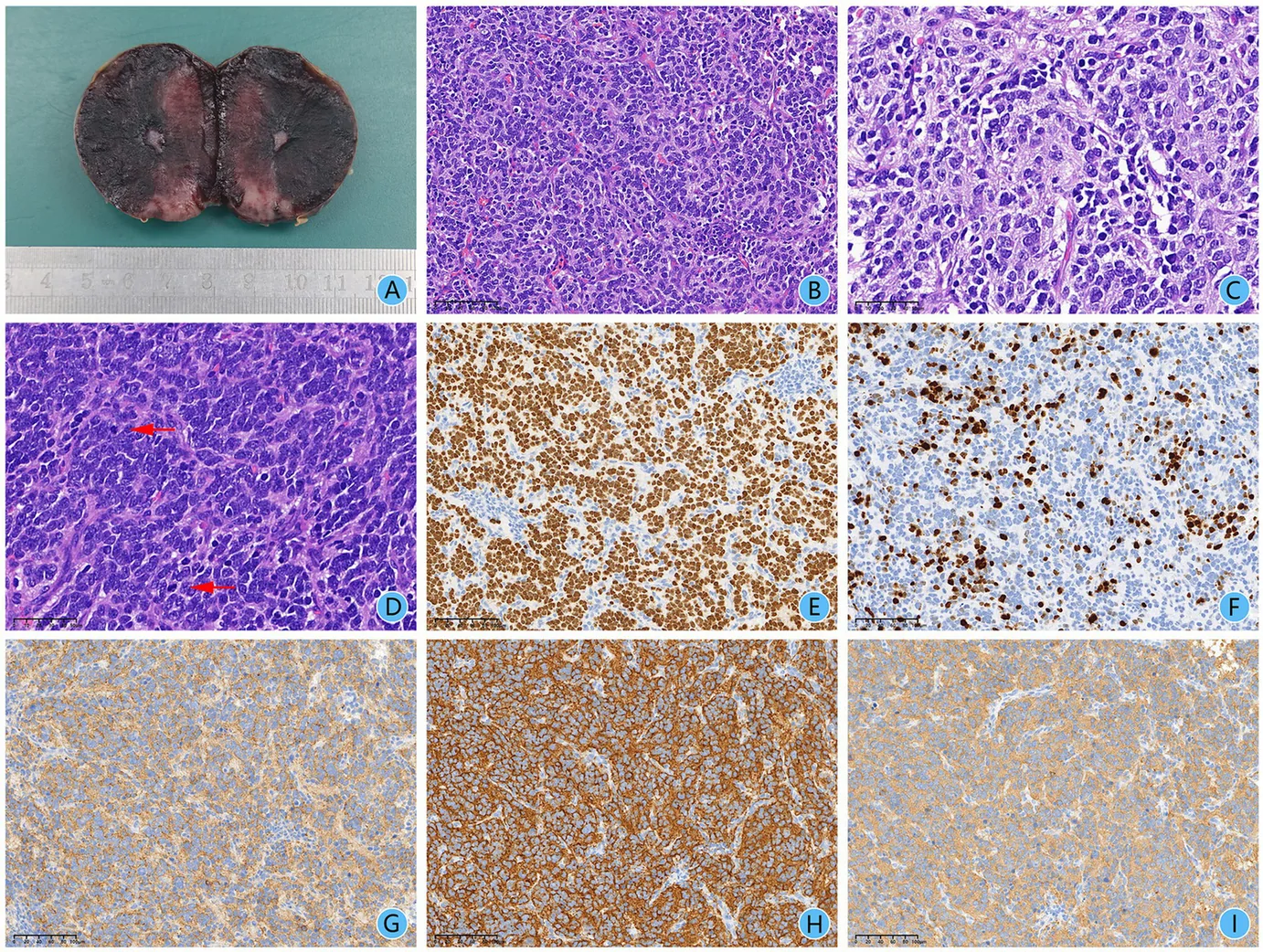
**(A)** Gross pathological specimen displaying a solid neuroblastoma characterized by a gray-red cut surface, soft consistency, and a fish-flesh appearance; **(B)** Histological section (H&E, ×200) showing a poorly differentiated tumor with neoplastic cells arranged in nests and nodules, separated by delicate fibrous vascular stroma; **(C)** Higher magnification (H&E, ×400) revealing neural meshwork structures within the background of the nodules; **(D)** Tumor cells exhibiting classic small round blue cell morphology, an increased nuclear-to-cytoplasmic ratio, and frequent mitotic figures (H&E, ×400); **(E)** Immunohistochemical staining for Phox2B demonstrating diffuse and strong positive expression (IHC, ×200); **(F)** Ki-67 proliferation index estimated at approximately 20% (IHC, ×200); **(G)** Chromogranin A (CgA) showing diffuse strong positive immunoreactivity (IHC, ×200); **(H)** Synaptophysin exhibiting diffuse strong positive expression (IHC, ×200); **(I)** Neuron-specific enolase (NSE) demonstrating diffuse strong positive staining (IHC, ×200).

**Figure 3:**
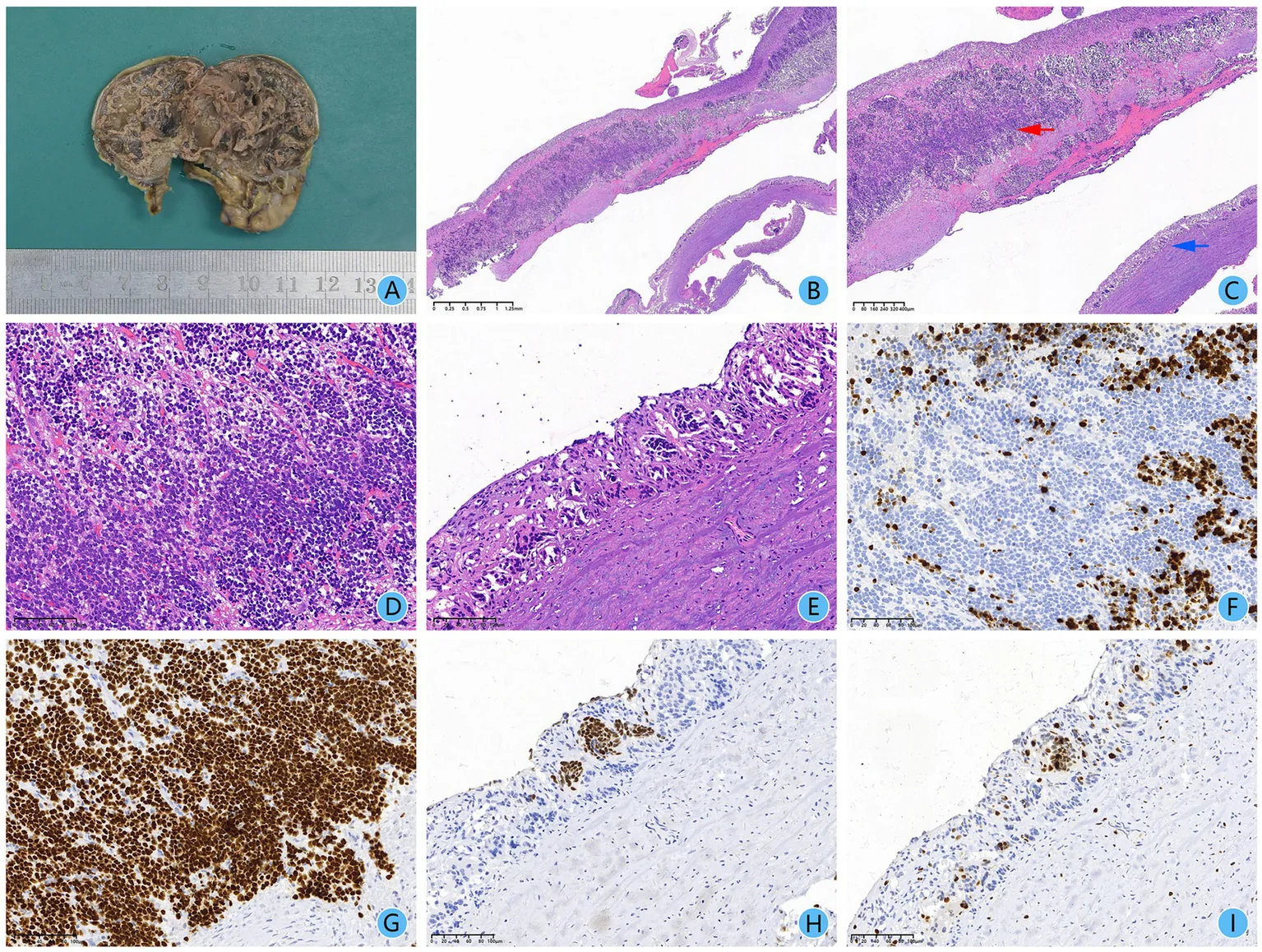
**(A)** Gross pathological specimen displaying an intact cystic neuroblastoma. A single cystic cavity was identified without obvious solid components. The cyst lumen contained blood clots and necrotic tissue, and the cyst wall was smooth with a thickness of approximately 0.1 cm–0.3 cm. **(B)** The cyst wall demonstrates a relatively thick composition predominantly of fibrous tissue (H&E, ×2). **(C)** At low magnification, small, blue, round tumor cells are arranged in nest-like or nodular patterns both within the cyst wall (indicated by the red arrow) and along the inner surface of the cyst wall (indicated by the blue arrow) (H&E, ×4). **(D)** High magnification reveals nest-like clusters of these small blue round tumor cells within the cyst wall (H&E, ×200). **(E)** Similarly, nodular aggregations of small blue round tumor cells are evident on the cyst’s inner wall at high magnification (H&E, ×200). **(F)** Immunohistochemical analysis shows a Ki-67 proliferation index of approximately 20% within the cyst wall (IHC, ×200). **(G)** Tumor cells located within the cyst wall exhibit positive for Phox2B (IHC, ×200). **(H)** Tumor cells on the cyst’s inner wall also demonstrate Phox2B positivity (IHC, ×200). **(I)** The Ki-67 proliferation index on the cyst inner wall is approximately 8% (IHC, ×200).

Gross examination of the 15 tumors revealed that 11 were solid and 4 were cystic. Tumor diameters ranged from 2.1 cm to 7.5 cm, with a mean diameter of 4.4 cm. The cut surfaces of solid tumors were gray-red, soft in consistency, and exhibited the characteristic fish-flesh appearance (Figure 2A). In cystic tumors, the cystic cavities contained turbid, dark red fluid; the cyst walls were smooth and measured approximately 0.1 cm to 0.3 cm in thickness (Figure 3A).

#### Histological analysis

In a cohort of 15 pediatric cases, histopathological evaluation identified 14 instances as poorly differentiated neuroblastoma, with one case classified as a composite neuroblastic tumor. Within this group, four cases represented distinct subtypes, specifically cystic neuroblastoma.

Microscopic examination of the poorly differentiated solid neuroblastomas revealed tumor cells organized into nests or nodules at low magnification, separated by delicate fibrovascular stroma (Figure 2B). A neuropil background was evident within these nodules (Figure 2C). The tumor cells displayed characteristic small blue round cell morphology, featuring a high nucleus-to-cytoplasm ratio and frequent mitotic (Figure 2D).

Cystic neuroblastoma predominantly manifested as cystic structures with relatively thick fibrous cyst walls (Figure 3B). Small blue round tumor cells were arranged in nests or nodules along the inner cyst wall (Figure 3C, red arrow) and within the cyst wall itself (Figure 3C, blue arrow). High magnification reveals nest-like clusters of these small blue round tumor cells within the cyst wall (Figures 3D,E).

**Figure 4:**
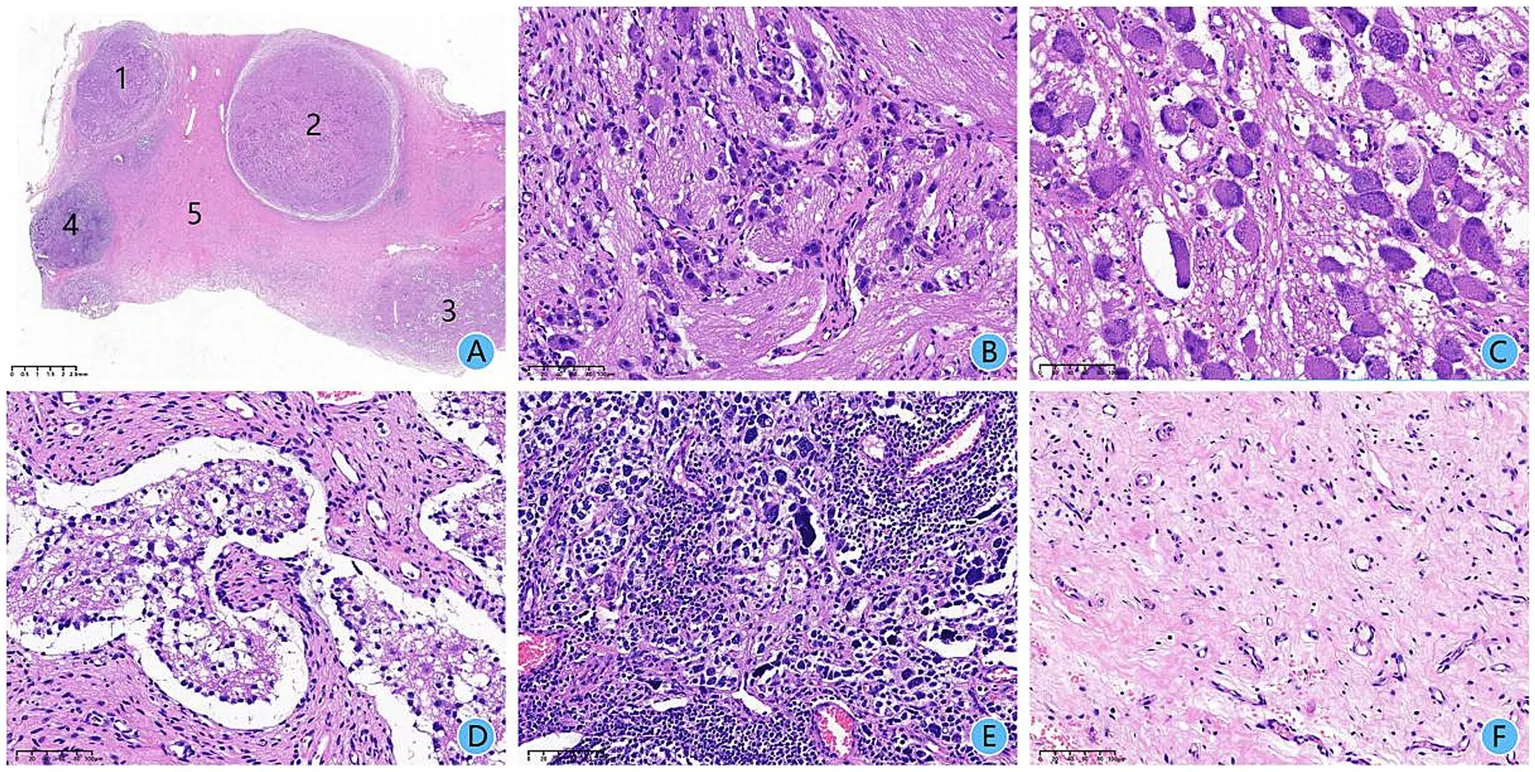
**(A)** Composite neuroblastoma composed of multiple nodules under the microscope; this section shows 4 nodules (labeled 1, 2, 3, 4) and the inter-nodular area (labeled 5) (H&E, ×0.6); **(B)** Nodule 1 showing differentiated neuroblastoma (H&E, ×200); **(C)** Nodule 2 showing mixed ganglionic neuroblastoma (H&E, ×200); **(D)** Nodule 3 also showing mixed ganglionic neuroblastoma (H&E,×200); **(E)** Nodule 4 showing poorly differentiated neuroblastoma with partial intercellular changes (H&E, ×200); **(F)** At high magnification, the inter-nodular area consists of fibrous stroma with no Schwann cell stroma observed (H&E, ×200).

In the sole case of composite neuroblastoma, multiple nodules were observed microscopically. As illustrated in Figure 4A, four nodules are present within the examined section, each containing diverse neuroblastoma components (Figures 4B–D). Certain regions exhibited anaplastic features (Figure 4E). The inter-nodular area (Figure 4A, marked area 5/Figure 4F) consisted of fibrous stroma, with an absence of Schwann cell stroma.

Assessment of proliferative activity indicated that, among the 15 cases, 10 exhibited a low mitosis-karyorrhexis index (MKI), 4 demonstrated a medium MKI, and 1 presented with a high MKI.

#### Immunohistochemical analysis

Immunohistochemical evaluation of tumor tissues from all 15 pediatric patients confirmed expression of neuroblastoma-specific markers (Figures 2E–I, 3F–I). Phox2B demonstrated diffuse and strong positivity across all cases. CgA and Syn were diffusely positive in 13 cases, while the remaining two cases showed focal positivity. Additional markers, including Ki67, NSE, S-100 and NF, were variably expressed (Table 3).

**Table 3:** Immunohistochemical staining results of the tumor specimens.

Patient No.	Phox2B	Syn	CgA	NSE	S-100	NF
1	+	+	−	Partial +	Partial +	−
2	+	+	+	Partial +	−	Partial +
3	+	+	−	Partial +	−	+
4	+	+	+	+	Partial +	Partial +
5	+	+	+	+	Partial +	+
6	+	+	Partial +	+	−	−
7	+	+	+	+	−	Not stained
8	+	+	Partial +	+	−	−
9	+	+	+	+	Partial +	−
10	+	Partial +	+	Partial +	Partial +	−
11	+	+	+	+	−	−
12	+	Partial +	+	Not stained	Not stained	−
13	+	+	+	Not stained	−	−
14	+	+	+	+	Partial +	−
15	+	+	+	+	+	+

#### Molecular genetic analysis

**Figure 5:**
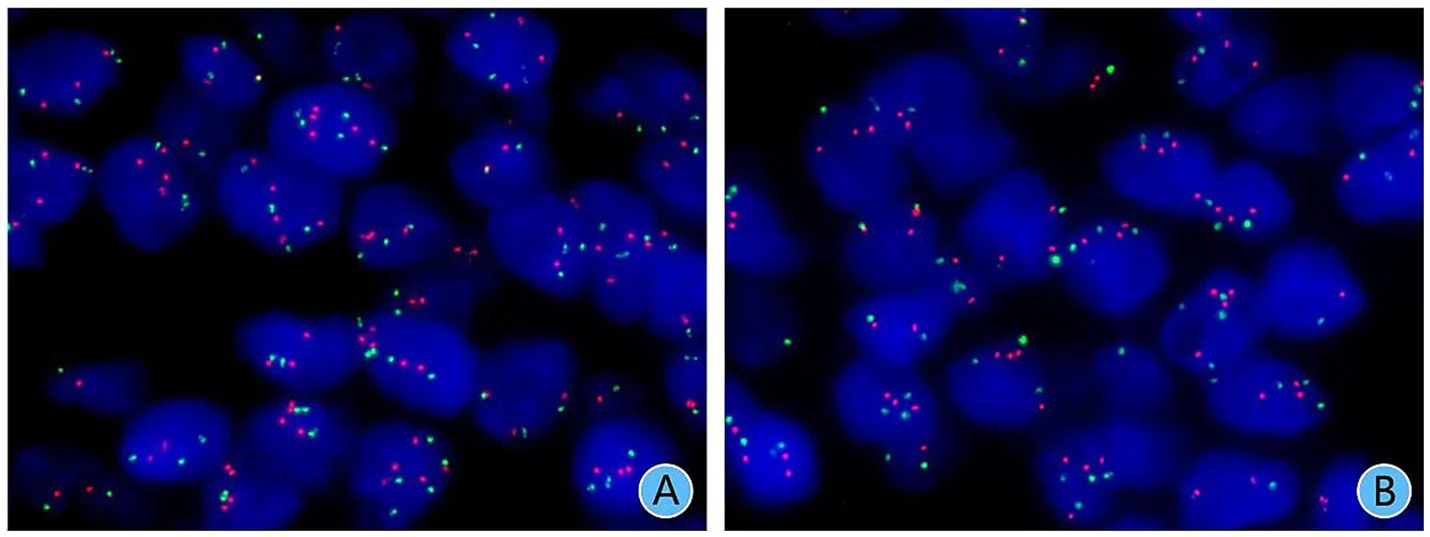
**(A)** *MYCN* gene amplification detection result shows gain (×1,000); **(B)** *KMT2A* (11q23) gene deletion detection result is normal (×1,000).

Among the 15 children in this group, 11 underwent *MYCN* gene amplification testing, of which 1 presented *MYCN* amplification ‘heterogeneous’, 8 showed *MYCN* gain, and 2 had normal *NMYC* status. As illustrated in Figure 5A, case 1 shows *MYCN* amplification ‘heterogeneous’, with amplification present in less than 10% of the tumor area and non-amplified cells detected in the remaining regions. This patient subsequently underwent whole-genome microarray analysis specific to pediatric neuroblastoma, which identified 20 acquired copy number variation (CNV) abnormalities, six of which were directly associated with neuroblastoma. Notably, no *MYCN* gene amplification was detected, nor was there any loss of heterozygosity (LOH) in the 1p or 11q chromosomal regions. Conversely, a gain was observed in the 17q11.2–q25.3 region. Chromosomal ploidy was near triploid, and the DNA copy number profile revealed only numerical chromosome aberrations (NCA), characterized by whole chromosome number changes. Among the 11 patients evaluated for *MYCN* amplification, 4 also underwent testing for *KMT2A* (11q23) gene deletion, with all results returning negative, as depicted in Figure 5B.

## Treatment

Among the 15 patients, only one, whose primary tumor was located in the left adrenal gland with concurrent hepatic metastasis, underwent postoperative chemotherapy in accordance with the high-risk protocol specified in the “Expert Consensus on Diagnosis and Treatment of Pediatric Neuroblastoma (2015)” (2). The remaining 14 patients did not receive any radiotherapy or chemotherapy either before or after surgical intervention. Based on the International Neuroblastoma Staging System (INSS) (3), the distribution of disease stages was as follows: Stage I in 12 patients, Stage II in 2 patients, and Stage IV in 1 patient.

## Follow-up

Follow-up assessments were conducted until October 30th, 2025. Two patients were lost to follow-up, while complete follow-up data were available for 13 patients, with durations ranging from 1 to 76 months and a median follow-up period of 10 months. The follow-up outcomes indicated that 12 children remained tumor-free survivors. However, one patient with a primary mediastinal tumor demonstrated a mediastinal shadow on computed tomography 6 months postoperatively, suggestive of tumor recurrence.

## Discussion

Neuroblastoma is a tumor distinguished by heterogeneous levels of neuroblast differentiation and diverse stages of Schwann cell stroma maturation. It arises from neuroectodermal cells of the neural crest and represents the most prevalent extracranial solid malignant neoplasm in pediatric populations (4). The term neonatal neuroblastoma specifically denotes cases diagnosed either prenatally or within the first 28 days postpartum. Data from the Children’s Oncology Group (COG) Statistics and Data Center indicate that neonatal neuroblastoma constitutes approximately 5% of all neuroblastoma incidences (5).

Our study included 15 pediatric patients diagnosed with congenital neuroblastoma. Benefiting from the advent and widespread application of prenatal diagnostic methodologies (5, 6), 8 of the cases were diagnosed via ultrasonography between 32 and 39 weeks of gestation. Consistent with Kostyrka B’s study (7), all lesions in these 8 cases were located in the adrenal glands. This percentage is considerably higher than that seen in conventional neuroblastoma, the incidence of which is approximately 50%. In addition to the eight cases detected prenatally mentioned above, our cohort included seven neonatal cases (within 28 days after birth). Among these patients, four lesions originated from the adrenal glands, and the remaining three arose from other sites, including the neck, retroperitoneum and posterior mediastinum. These distribution characteristics of primary lesions were consistent with previous study (8–10). More interestingly, all our cystic neuroblastoma cases were adrenal in origin. Some researches (11, 12) posit that the development of perinatal neuroblastoma and its cystic variant may be associated with nodular aggregations of neuroblasts during adrenal gland ontogeny.

Accurate pathological identification is essential for the diagnosis and subtyping of congenital neuroblastoma. According to the 5th Edition of the WHO Classification of Pediatric Tumors (13), a combined immunohistochemical panel covering neuronal and neural crest biomarkers, including phox2B, Syn, NF and CgA, is recommended to assist pathological diagnosis. All of our 15 cases expressed diffuse and strong signals of Phox2B in immunohistochemical examination, as its highest diagnostic specificity among the above markers, it maintains stable immunoreactivity even in undifferentiated neuroblastoma subtypes (14, 15). Syn and CgA were diffusely positive in 13 cases with other 2 cases showed focal positivity. NF were variably expressed. Moreover, S100 protein can specifically label Schwannian stromal components, which facilitates the differentiation of neuroblastoma from other neuroblastic neoplasms with similar morphological features, and acts as a supplementary indicator for pathological differential diagnosis (16). Overall, the immunohistochemical expression patterns of these biomarkersaligned with the diagnostic criteria established by the WHO and previous studies (14–16).

Amplification of the *MYCN* oncogene is closely linked to the aggressive biological behavior of tumors and represents the most significant independent prognostic risk factor within the neuroblastoma risk stratification framework (17, 18). The Neuroblastoma Expert Panel of the National Comprehensive Cancer Network (NCCN) Clinical Practice Guidelines in Oncology advocates for the assessment of *MYCN* amplification status in all neuroblastoma cases, including the neuroblastoma nodule components found in nodular ganglioneuroblastoma (19). Among segmental chromosomal aberrations in neuroblastoma, deletion of genetic material on the long arm of chromosome 11 (11q), is the most extensively characterized and serves as an independent prognostic indicator of poor outcome (20, 21). In patients aged over 18 months, the presence of segmental chromosomal aberrations, particularly 11q deletion, correlates with reduced event-free survival and overall survival rates (22). The Biology Committee of the International Neuroblastoma Risk Group (INRG) recommends that FISH testing for the *MYCN* and *KMT2A* genes be performed in neuroblastoma (23). In the current investigation, none of the 11 cases of congenital neuroblastoma demonstrated amplification of the *MYCN* gene, and analysis of the *KMT2A* (11q23) gene in four cases yielded negative results. These observations suggest potential molecular differences between this subtype and conventional neuroblastoma. Nevertheless, the relatively small sample size of 15 cases constitutes a limitation that may impact the generalizability of the findings.

In the case presented herein (Case 15), the primary tumor demonstrated progressive enlargement over a 22-month follow-up period, culminating in complete surgical excision. Histopathological analysis revealed a heterogeneous composition comprising poorly differentiated neuroblastoma (with some anaplastic cells), differentiating neuroblastoma, mixed-type ganglioneuroblastoma, and mature intermediate-type ganglioneuroma, accompanied by synchronous cervical lymph node metastasis. Partial separate tumor nodules exhibited a marked deficiency in Schwannian stroma, consistent with those reported in the existing literature (24, 25). This tumor entity encompasses a distinct subtype known as composite neuroblastoma, classified under nodular neuroblastoma (Neuroblastoma, nodular, NBn). Its defining features include the presence of multiple well-demarcated nodular lesions within the tumor, each representing components at varying stages of neuroblast differentiation (e.g., poorly differentiated, differentiating). Unlike conventional nodular neuroblastoma, the overall tumor background is notably deficient in Schwannian stroma, and the tumor cells within discrete nodules derive from clonally distinct populations exhibiting marked biological heterogeneity. Some scholars (26, 27) advocated for the inclusion of composite neuroblastoma in forthcoming revisions of the International Neuroblastoma Pathology Classification (INPC). According to extant literature (24, 25), this multi-component histological pattern aligns with the “age-related maturation sequence” characteristic of NBn, wherein distinct clonal components correspond to various stages of differentiation and maturation during tumor progression. The cervical lymph node metastasis observed in Case 15 indicates biologically aggressive intratumoral clones. This observation is congruent with the clinical and pathological paradigm of NBn, wherein prognosis is predominantly influenced by invasive clones. Although Case 15 lacked *MYCN* amplification and *KMT2A* (11q23) abnormalities and exhibited a low MKI, the presence of anaplastic nodules may account for the tumor’s aggressive biological behavior.

This study demonstrates that serum NSE serves as a relatively reliable preoperative diagnostic biomarker for congenital neuroblastoma, whereas the diagnostic utility of VMA is comparatively limited. Nonetheless, the efficacy of NSE as a sole indicator for early detection of postoperative recurrence is constrained. Transient postoperative elevations or dynamic fluctuations in NSE levels may represent nonspecific pathophysiological responses to tumor resection rather than definitive markers of recurrence. Furthermore, normal NSE concentrations do not preclude the possibility of recurrence, as evidenced by cases in this study where NSE levels remained within normal ranges despite tumor relapse. Consequently, clinical diagnosis and management should clearly differentiate the diagnostic roles of these two biomarkers. Postoperative surveillance should move beyond reliance on a single biomarker and instead implement a comprehensive monitoring strategy that integrates regular serum NSE assessments with imaging modalities such as radiological imaging examinations. This multifaceted approach can mitigate the limitations inherent to NSE measurement, facilitate earlier detection of recurrence risk, and ultimately enhance long-term outcomes in pediatric patients.

Importantly, dynamic alterations in serum NSE levels and imaging findings exhibit a strong correlation with disease status, serving as reliable indicators for monitoring postoperative recovery and early recurrence risk. Consequently, these parameters represent essential components of clinical follow-up protocols for congenital neuroblastoma. Overall, the prognosis for congenital neuroblastoma appears favorable; most affected patients do not require adjuvant radiotherapy or chemotherapy following surgical resection but instead undergo long-term standardized clinical surveillance (28). This single-center retrospective study enrolled 15 children diagnosed with congenital neuroblastoma, and postoperative tumor recurrence was observed in only one case, with a limited sample size. Our results indicate that serum NSE alone cannot reliably predict tumor recurrence, whereas the combination of NSE and imaging examinations is promising for postoperative prognostic evaluation and recurrence surveillance in these pediatric patients. Further large-sample, multicenter clinical studies are required to validate this conclusion and establish standardized postoperative follow-up protocols.

## Data Availability

The original contributions presented in the study are included in the article/supplementary material, further inquiries can be directed to the corresponding author.
